# Association between HLA genes and American cutaneous leishmaniasis in endemic regions of Southern Brazil

**DOI:** 10.1186/1471-2334-13-198

**Published:** 2013-05-02

**Authors:** Rejane C Ribas-Silva, Adriana D Ribas, Maria CG dos Santos, Waldir V da Silva, Maria VC Lonardoni, Sueli D Borelli, Thaís GV Silveira

**Affiliations:** 1 Postgraduate Program in Health Science, Universidade Estadual de Maringá (UEM), Maringá, Paraná, Av. Colombo, 5.790 - Jd. Universitário, 87020-900, Brazil; 2 Department of Biostatistics, Universidade Estadual de Maringá (UEM), Maringá, Paraná, Av. Colombo, 5.790 - Jd. Universitário, 87020-900, Brazil; 3 Leishmaniases Laboratory, Department of Clinical Analyses and Biomedicine, Universidade Estadual de Maringá (UEM), Maringá, Paraná, Av. Colombo, 5.790 - Jd. Universitário, 87020-900, Brazil; 4 Immunogenetics Laboratory, Department of Basic Health Sciences, Universidade Estadual de Maringá (UEM), Maringá, Paraná, Av. Colombo, 5.790 - Jd. Universitário, 87020-900, Brazil

**Keywords:** Genetic susceptibility, HLA, Leishmaniasis, *Leishmania brasiliensis*

## Abstract

**Background:**

The present study sought to investigate the association between HLA-A, HLA-B and HLA-DRB1 genes and susceptibility or resistance to the different clinical manifestations of American cutaneous leishmaniasis (ACL) in southern Brazil.

**Methods:**

The sample consisted of 169 patients with a diagnosis of ACL and 270 healthy subjects for comparison. HLA-A, HLA-B and HLA-DRB1 were typed by PCR-SSO reverse dot blot.

**Results:**

Results showed a trend towards susceptibility to cutaneous lesions for alleles HLA-DRB1*13 (*P*=0.0228; *Pc*=0.3420; OR=1.66; 95%CI=1.08 – 2.56), HLA-B*35 (*P*=0.0218; *Pc*=0.6758; OR=1.67; 95%CI=1.08 – 2.29) and HLA-B*44 (*P*=0.0290; *Pc*=0.8990; OR=1.67; 95%CI=1.05 – 2.64). Subjects with allele HLA-B*27 (*P*=0.0180; *Pc*=0.5580; OR=7.1111; 95%CI=1.7850 – 28.3286) tended towards susceptibility to mucocutaneous lesions, those with HLA-B*49 (*P*=0.0101; *Pc*=0.3131; OR=6.4000; 95%CI=1.8472 – 22.1743) to recurrent ACL, and HLA-B*52 (*P*=0.0044; *Pc*=0.1360; OR=12.61; 95%CI=3.08 – 51.66), to re-infection. Presence of HLA-B*45 (*P*=0.0107; *Pc*=0.3317) tended to provide protection against the cutaneous form of ACL. The most frequent haplotypes that may be associated with susceptibility to ACL were A*02 B*44 DRB1*07 (*P* = 0.0236) and A*24 B*35 DRB1*01 (*P* = 0.0236).

**Conclusion:**

Some Class I and Class II HLA genes appear to contribute towards susceptibility to and protection against different clinical manifestations of ACL. Other genetic marker studies may contribute toward future prophylactic and therapeutic interventions in ACL.

## Background

American cutaneous leishmaniasis (ACL) is a non-contagious infectious disease, caused by several species of protozoa in the genus *Leishmania* and transmitted by sandfly bites, which causes skin and mucosal lesions [1-3].

In Brazil, ACL has been notified to the health authorities since the 1980s. Between 1990 and 2008, some 527,976 cases were notified, 12,115 of which in the southern region of Brazil, with 11,557 cases (95.4%) from the state of Paraná alone [4]. The northern and western regions of the Southern state of Paraná stand out due to their high concentration of ACL cases, and are thus considered areas of epidemiological importance [5]. In these regions, the predominant species (98.7%) is *Leishmania (Viannia) braziliensis*[6,7].

Infection by *L. braziliensis* may develop into the localized clinical cutaneous and/or mucosal forms of ACL. Cure of ACL is determined clinically, and is defined by regression of lesions within three months of concluding therapy. Periodical follow-up for 12 months is recommended. However, relapse of the disease may occur during the first year after cure. Relapse consists of the appearance of the cutaneous lesion at its former site or recurrence of mucosal lesions due to potential hematogenous spread of the infection [3]. It is estimated that relapse of cutaneous lesions occurs in approximately 10% of infected patients, whereas mucocutaneous lesion relapse, which constitutes the most severe complication of ACL, occurs in 4% of cases [8-11]. A distinct manifestation is recurrent ACL, which, unlike relapse, consists of the onset of cutaneous lesions in places other than those of the previous infection, possibly indicating reinfection [10].

The progression of ACL varies according to environmental factors, vector characteristics, parasite genetics and host immunological factors. Furthermore, patients infected with the same *Leishmania* species may develop different clinical forms of ACL even if they have the same nutritional conditions and are exposed to the same environmental factors [12-15]. In certain studies, HLA alleles have been associated with disease progression in cutaneous and mucocutaneous leishmaniasis [16-19]. In this context, the conduction of studies related to host genetic markers after exposure to the parasite is highly relevant.

Due to the dearth of data on genetic polymorphism of the HLA system and its association with protection against and/or susceptibility to development of ACL in the Brazilian population, the present study investigated possible involvements of Class I (HLA-A, HLA-B) and Class II (HLA-DRB1) HLA genes in the different clinical manifestations of ACL in populations of endemic regions in Southern Brazil where *L. braziliensis* is the predominant parasite.

## Methods

### Patients and controls

A retrospective study was carried out on the epidemiological records of patients from the 13^th^ and 15^th^ Health Sections of the state of Paraná diagnosed with ACL. The diagnosis was made at the Leishmaniases Laboratory of the State University of Maringá. Patients with clinical manifestations of the disease and with positive parasite detection and/or Montenegro skin test who received a medical prescription for treatment two years ago were selected. This period was defined because, according to WHO, more than 90% of recurrent cases occur up to one year after treatment.

Patient age at data collection ranged between 17 and 83 years (mean, 47.38 ± 14.79); 139 (82.3%) patients were males and 30 (17.8%) were females. According to ethnicity (skin color using Brazilian census categories), patient groups consisted of 127 (75.2%) caucasian; 36 (21.3%) amerindians; 4 (2.4%) african and 2 (1.2%) asian.

The control group comprised 260 healthy subjects, chosen according to age, gender, ethnicity, occupation and other demographic parameters, who had visited areas where ACL is endemic and had no clinical manifestations of the disease. Age ranged between 18 and 73 years (mean, 30.31 ± 10.92); 141 controls (54.2%) were males and 119 (45.8%) were females. The ethnic distribution was 208 (80.0%) white, 37 (14.2%) brown, 8 (3.1%) black and 7 (2.7%) yellow.

Research subjects were interviewed and information was recorded in social-epidemiological files which included notes on clinical manifestations occurring after treatment, such as development of the mucosal form or even relapse. All subjects were informed of the investigation, and those who agreed to allow use of their data provided written informed consent. The study was approved by the Permanent Human Subject Research Ethics Committee of the State University of Maringá, with judgment number 153/2009.

### Group composition

The study population comprised 169 subjects with a positive diagnosis of ACL who had received therapy and lived in Southern Brazil. The control group consisted of 260 subjects with no clinical manifestations of ACL who lived in the same area from which patients were recruited and who also frequented areas posing a high risk of infection (shrubland with nearby streams). Subjects belonging to both groups were unrelated.

**Figure 1:**
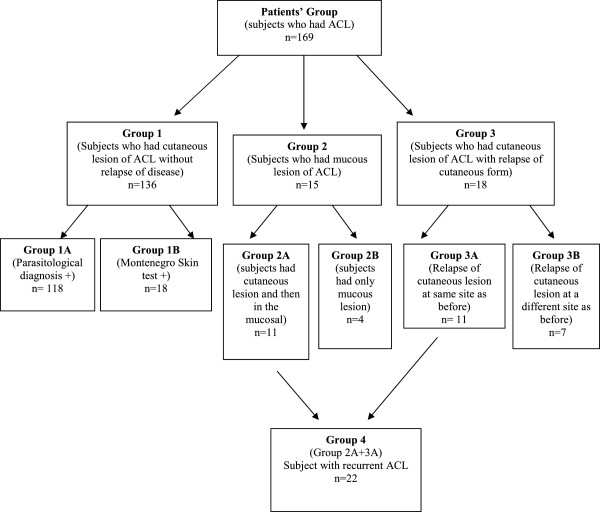
Composition of groups and subgroups comprising ACL group (n=169).

According to clinical and laboratory data, patients were allocated into 4 groups and 6 subgroups (Figure 1).

Group 1 comprised subjects who had a cutaneous form of the disease and no evidence of any relapse. This group was subdivided into Group 1A, composed of 118 individuals who had a positive parasitological diagnosis, and Group 1B, composed of 18 individuals who had only a positive Montenegro test.

Group 2 consisted of subjects with clinical manifestations of mucosal disease. This group was subdivided into Group 2A, composed of 11 individuals with a positive laboratory diagnosis (parasite survey and/or Montenegro skin test) of a past cutaneous lesion with subsequent development of a mucosal lesion, and Group 2B, comprising 4 individuals who had a mucosal lesion but no history of previous cutaneous lesions.

Group 3 comprised individuals who had the cutaneous form of the disease and, after treatment, developed cutaneous lesions again. This group was subdivided into Group 3A, composed of 11 individuals who had a positive laboratory diagnosis (parasitological and/or Montenegro skin test) and lesion relapse at the original site, and Group 3B, composed of 7 individuals who had a positive laboratory diagnosis (parasitological and/or Montenegro skin test) and lesion relapse at a different site.

Group 4 comprised all subjects with a history of recurrent disease—Groups 2A and 3A. Recurrent ACL was defined as the onset of a new lesion after at least 1 year of treatment, whether at the same site of the previous lesion (Group 3A) or, subsequently, in the mucosa (Group 2A).

The small number of individuals in certain groups and subgroups of ACL was a limiting factor in this study, justified by the frequency of some clinical manifestations. Recurrence of ACL occurs in approximately 10% of infected patients, and mucocutaneous lesion relapse occurs in 4% of infected patients [8-11]. Thus, this study demonstrates the preliminary results of these groups.

### Determination of HLA alleles

Peripheral blood samples (10 mL) were collected from patients and controls into EDTA anticoagulant tubes and centrifuged at 960 g for 10 minutes. Nucleated cells were separated and frozen at -80°C until use. Genomic DNA was extracted from 100μL aliquots of frozen cells with the EZ-DNA extraction kit (Biological Industries®, Kibbutz Beit Haemek, Israel), according to manufacturer instructions.

Patients’ and controls’ HLA Class I (A, B) and Class II (DRB1) genotypes were determined by the reverse dot blot PCR-SSO method, using the commercially available RELI™ SSO kit (DYNAL BIOTECH A.S.A, Oslo, Norway). Genotyping was performed at the Immunogenetic Laboratory of the State University of Maringá.

### Statistical analysis

A data bank of HLA-A, -B and –DRB1 processing results was created in Microsoft Excel 2007. Data were expressed as allele frequency (*F*), calculated as the ratio of the number of times different alleles appeared in the sample to the total number of alleles. The *P*-value was calculated using a two-tailed Fisher’s exact test at a 5% significance level. Significant *P*-values were corrected with the Bonferroni method (*P*c) by multiplying *P* by the number of alleles detected for each locus. Again, *P*c values <0.05 were considered significant. Odds ratios (OR) at a 95% confidence interval (CI) were calculated for results with *P* < 0.05. Whereas descriptive statistical analyses for sex and ethnicity variables were determined by Fisher’s exact test, the age variable was calculated with the *t*-test for independent samples, also with a significance level of 5%. Statistical analyses were performed in the *R* and Statistica 7.0 software packages. Arlequin 2000 http://cmpg.unibe.ch/software/arlequin3/ was employed to confirm the distribution of allele frequency and *P* > 0.05 rates were in Hardy–Weinberg equilibrium (HW). Haplotype frequencies were estimated using the Expectation Maximization (EM) algorithm included in the Arlequin software.

## Results

The present study investigated a potential association between ACL and Class I and Class II HLA alleles in a Southern Brazilian population by comparing the frequency of HLA-A, HLA-B and HLA-DRB1 alleles in subjects affected by the disease and those of healthy controls.

The distribution of allele proportions reported is in HW equilibrium both in the patient group and in the control one.

### ACL overall versus control group

Table 1 shows the HLA-A, HLA-B and HLA-DRB1 allele frequencies of the group. Overall, there were differences between ACL patients and the control group for allele HLA-B*45, which was more frequent in the control group [0% vs. 2.31% (*P* = 0.0046 and *Pc* = 0.1426)], with a trend towards resistance to ACL development. The presence of HLA-DRB1*13 in the ACL and control groups [15.1% vs. 10.38% (*P* = 0.0431; *Pc* = 0.6465; OR = 1.5335; 95%CI = 1.0177 – 2.3107)] suggested a trend towards ACL susceptibility.

**Table 1:** **Frequency of HLA-A, -B and -DRB1 alleles in the ACL group *versus *the control group**

	**ACL (n = 169)**		**Controls (n = 260)**							**ACL (n = 169)**		**Controls (n = 260)**					
**Allele**	**n**	***F *%**	**n**	***F %***	***P*-value**	**Pc-value**	**OR**	**95%CI**	**Allele**	**n**	***F%***	**n**	***F%***	***P*-value**	**Pc-value**	**OR**	**95%CI**
HLA – A*									HLA-B*								
**01**	37	10.9	47	9.0	ns	-			**07**	20	5.9	38	7.3	ns	-		
**02**	95	28.1	127	24.4	ns	-			**08**	16	4.7	34	6.5	ns	-		
**03**	30	8.8	56	10.8	ns	-			**13**	1	0.3	9	1.7	ns	-		
**11**	23	6.8	26	5.0	ns	-			**14**	15	4.4	22	4.2	ns	-		
**23**	18	5.3	22	4.2	ns	-			**15**	26	7.7	48	9.2	ns	-		
**24**	37	10.9	62	11.9	ns	-			**18**	17	5.0	36	6.9	ns	-		
**25**	4	1.2	13	2.5	ns	-			**27**	7	2.1	8	1.5	ns	-		
**26**	11	3.2	18	3.5	ns	-			**35**	58	17.2	68	13.1	ns	-		
**29**	9	2.6	28	5.4	ns	-			**37**	1	0.3	5	1.0	ns	-		
**30**	11	3.2	25	4.8	ns	-			**38**	10	3.0	15	2.9	ns	-		
**31**	18	5.3	19	3.7	ns	-			**39**	13	3.9	18	3.5	ns	-		
**32**	8	2.3	16	3.1	ns	-			**40**	18	5.3	16	3.1	ns	-		
**33**	5	1.5	15	2.9	ns	-			**41**	2	0.6	6	1.2	ns	-		
**34**	3	0.9	5	1.0	ns	-			**42**	2	0.6	6	1.2	ns	-		
**36**	2	0.6	2	0.4	ns	-			**44**	43	12.7	46	8.9	ns	-		
**39**	1	0.3	-	-	ns	-			**45**	0	-	12	2.3	0.0046	0.1426	-	-
**66**	3	0.9	1	0.2	ns	-			**46**	0	-	4	0.8	ns	-		
**68**	19	5.62	35	6.7	ns	-			**47**	0	-	1	0.2	ns	-		
**74**	4	1.9	3	0.6	ns	-			**48**	3	0.9	2	0.4	ns	-		
HLA-DRB1*									**49**	8	2.4	8	1.5	ns	-		
**01**	23	6.8	42	8.1	ns	-			**50**	8	2.4	14	2.7	ns	-		
**03**	32	9.5	61	11.7	ns	-			**51**	29	8.6	44	8.5	ns	-		
**04**	40	11.8	56	10.8	ns	-			**52**	10	3.0	11	2.1	ns	-		
**07**	39	11.5	67	12.9	ns	-			**53**	7	2.1	8	1.5	ns	-		
**08**	26	7.7	30	5.8	ns	-			**54**	1	0.3	3	0.6	ns	-		
**09**	4	1.2	4	0.8	ns	-			**55**	8	2.4	12	2.3	ns	-		
**10**	5	1.5	10	1.9	ns	-			**56**	2	0.6	1	0.2	ns	-		
**11**	45	13.3	81	15.6	ns	-			**57**	7	2.1	12	2.3	ns	-		
**12**	3	0.9	10	1.9	ns	-			**58**	6	1.8	10	1.9	ns	-		
**13**	51	15.1	54	10.4	0.0431	0.6465	1.53	1.01-2.31	**73**	0	-	1	0.2	ns	-		
**14**	19	5.6	40	7.7	ns	-			**81**	0	-	2	3.9	ns	-		
**15**	34	10.1	44	8.8	ns	-											
**16**	17	5.0	21	4.0	ns	-											

n = number of times the allele occurs; F = allele frequency; *P*-value = calculated by Fisher’s exact test; Pc-value = value of p corrected by Bonferroni correction; OR = odds ratio; 95%CI = 95% confidence interval; ns= not significant (*p* > 0.05).

Haplotype analysis revealed frequency of 772 haplotypes in the ACL group and 1118 in the control group. Only haplotypes whose frequencies appeared to differ between groups (17 haplotypes) were analyzed. There were statistical differences between patients with ACL and controls for the haplotypes A*02 B*44 DRB1*07 (2.37% vs. 0% [*P* = 0.0236]) and A*24 B*35 DRB1*01 (2.37% vs. 0% [*P* = 0.0236]). Thus, these two haplotypes may be involved in susceptibility to ACL (Table 2).

**Table 2:** **Frequency of HLA-A/-B/–DRB1 haplotypes in the ACL group *versus *the control group**

**Haplotypes**	**Frequency (%)**			
	**Controls (n=260)**	**ACL (n=169)**	***P*-value**	**OR (95%CI)**
HLA-A*02-B*44-DRB1*07	0 (0.00%)	4 (2.37%)	0.0236	-
HLA-A*24-B*35-DRB1*01	0 (0.00%)	4 (2.37%)	0.0236	-

*P*-value obtained by Fisher's exact test. Of all analyzed haplotypes, only those having *P*-values <0.05 were added to this table.

### Group 1 *versus* control group

Table 3 shows a comparison of HLA-A, HLA-B and HLA-DRB1 allele frequencies between Group 1 and the control group. Differences exist between Group 1 and the control group for HLA-B*35, which was more frequent in patients [19.5% vs. 13.1% (*P* = 0.0218; *Pc* = 0.6758; OR = 1.6086; 95%CI = 1.0851 – 2.3848), and HLA-B*44 [13.9% vs. 8.8% (*P* = 0.0290; Pc = 0.8990; OR = 1.6734; 95%CI = 1.0592 – 2.6436), which suggests susceptibility to the cutaneous form of ACL. HLA-B*45 was only present in controls [0% vs. 2.31% (*P* = 0.0107; *Pc* = 0.3317)], which suggests that it may confer resistance to the cutaneous form of ACL. Moreover, comparison of HLA-DRB1*13 frequency in group 1 versus controls [16.2% vs. 10.4% (*P* = 0.0228; *Pc* = 0.3420; OR = 1.6654; 95%CI = 1.0851 – 2.5561) indicated a possible susceptibility to the cutaneous form of ACL.

**Table 3:** Frequency of HLA-A, -B and –DRB1 alleles in ACL Group 1 and the control group

	**ACL (group 1) (n = 136)**		**Controls (n = 260)**							**ACL (group 1) (n = 136)**		**Controls (n = 260)**					
**Allele**	**n**	***F *%**	**n**	***F %***	***P*-value**	***P*c-value**	**OR**	**95%CI**	**Allele**	**n**	***F %***	**n**	***F%***	***P*-value**	***P*c–value**	**OR**	**95%CI**
HLA – A*									HLA-B*								
**01**	29	10.7	47	9.0	ns	-			**07**	15	5.5	38	7.3	ns	-		
**02**	73	26.8	127	24.4	ns	-			**08**	10	3.7	34	6.5	ns	-		
**03**	24	8.8	56	10.8	ns	-			**13**	1	0.4	9	1.7	ns	-		
**11**	18	6.6	26	5.0	ns	-			**14**	14	5.2	22	4.2	ns	-		
**23**	14	5.2	22	4.2	ns	-			**15**	23	8.5	48	9.2	ns	-		
**24**	28	10.3	62	11.9	ns	-			**18**	13	4.8	36	6.9	ns	-		
**25**	4	1.5	13	2.5	ns	-			**27**	3	1.1	8	1.5	ns	-		
**26**	8	2.9	18	3.5	ns	-			**35**	53	19.5	68	13.1	0.0218	0.6758	1.67	1.08-2.29
**29**	9	3.3	28	5.4	ns	-			**37**	1	0.4	5	1.0	ns	-		
**30**	9	3.3	25	4.8	ns	-			**38**	7	2.6	15	2.9	ns	-		
**31**	15	5.5	19	3.7	ns	-			**39**	10	3.7	18	3.5	ns	-		
**32**	7	2.6	16	3.1	ns	-			**40**	14	5.2	16	3.1	ns	-		
**33**	5	1.8	15	2.9	ns	-			**41**	2	0.7	6	1.2	ns	-		
**34**	3	1.1	5	1.0	ns	-			**42**	2	0.7	6	1.2	ns	-		
**36**	1	0.4	2	0.4	ns	-			**44**	38	14.0	46	8.9	0.0290	0.8990	1.67	1.05-2.64
**39**	1	0.4	-	-	ns	-			**45**	0	-	12	2.3	0.0107	0.3317	-	-
**66**	2	0.7	1	0.2	ns	-			**46**	0	-	4	0.8	ns	-		
**68**	19	7.0	35	6.7	ns	-			**47**	0	-	1	0.2	ns	-		
**74**	3	1.1	3	0.6	ns	-			**48**	3	1.1	2	0.4	ns	-		
HLA-DRB1*									**49**	4	1.5	8	1.5	ns	-		
**01**	18	6.6	42	8.1	ns	-			**50**	6	2.2	14	2.7	ns	-		
**03**	22	8.1	61	11.7	ns	-			**51**	24	8.8	44	8.5	ns	-		
**04**	33	12.1	56	10.8	ns	-			**52**	6	2.2	11	2.1	ns	-		
**07**	33	12.1	67	12.9	ns	-			**53**	7	2.6	8	1.5	ns	-		
**08**	20	7.4	30	5.8	ns	-			**54**	0	-	3	0.6	ns	-		
**09**	4	1.5	4	0.8	ns	-			**55**	5	1.8	12	2.3	ns	-		
**10**	5	1.8	10	1.9	ns	-			**56**	1	0.3	1	0.2	ns	-		
**11**	33	12.1	81	15.6	ns	-			**57**	5	1.8	12	2.3	ns	-		
**12**	3	1.1	10	1.9	ns	-			**58**	5	1.8	10	1.9	ns	-		
**13**	44	16.2	54	10.4	0.0228	0.3420	1.66	1.08-2.55	**73**	0	-	1	0.2	ns	-		
**14**	14	5.2	40	7.7	ns	-			**81**	0	-	2	3.9	ns	-		
**15**	28	10.3	44	8.8	ns	-											
**16**	15	5.5	21	4.0	ns	-											

n = number of times the allele occurs; F = allele frequency; *P*-value = calculated by Fisher’s exact test; Pc-value = value of *P* corrected by Bonferroni correction; OR = odds ratio; 95%CI = 95% confidence interval; ns= not significant (*p* > 0.05).

### Subgroup 1A *versus* control group

Table 4 shows HLA-A, HLA-B and HLA-DRB1 allele frequencies in subgroup 1A and the control group. When allele frequencies were compared between group 1A and the control group, HLA-B*44 [14.8% vs. 8.8% (*P* = 0.0160; *Pc* = 0.4960; OR = 1.7943; 95%CI = 1.1219 – 2.8696)] suggested a possible susceptibility to the cutaneous form of ACL. On the other hand, HLA-B*45 [0% vs., 2.31% (*P* = 0.0225; *Pc* = 0.6975) suggested possible resistance to the cutaneous form of ACL. HLA-DRB1*13 [16.10% vs. 10.38% (*P* = 0.0152; *Pc* = 0.2280; OR = 1.6562; 95%CI = 1.0591 – 2.5899) may also be involved in susceptibility to the cutaneous form of ACL.

**Table 4:** Frequency of HLA-A, -B and –DRB1 alleles in ACL subgroup 1A and control group

	**ACL (group 1A) (n = 118)**		**Controls (n = 260)**							**ACL (group 1A) (n = 118)**		**Controls**					
**Allele**	**n**	***F *%**	**n**	***F %***	***P*-value**	***P*c-value**	**OR**	**95%CI**	**Allele**	**n**	***F %***	**n**	***F %***	***P*-value**	***P*c-value**	**OR**	**95%CI**
HLA – A*									HLA-B*								
**01**	26	11.0	47	9.0	ns	-			**07**	14	5.9	38	7.3	ns	-		
**02**	64	27.1	127	24.4	ns	-			**08**	10	4.2	34	6.5	ns	-		
**03**	21	8.9	56	10.8	ns	-			**14**	10	4.2	22	4.2	ns	-		
**11**	16	6.8	26	5.0	ns	-			**15**	22	9.3	48	9.2	ns	-		
**24**	24	10.2	62	11.9	ns	-			**18**	10	4.2	36	6.9	ns	-		
HLA-DRB1*									**27**	3	1.3	8	1.5	ns	-		
**01**	16	6.8	42	8.1	ns				**35**	44	18.6	68	13.1	ns	-		
**03**	20	8.4	61	11.7	ns	-			**39**	8	3.4	18	3.5	ns	-		
**04**	27	11.4	56	10.8	ns	-			**40**	12	5.1	16	3.1	ns	-		
**07**	29	12.3	67	12.9	ns	-			**44**	35	14.8	46	8.9	0.0160	0.4969	1.79	1.12-2.87
**08**	17	7.2	30	5.8	ns	-			**45**	0	-	12	2.3	0.0231	0.0225	-	-
**11**	31	13.1	81	15.6	ns	-			**49**	3	1.3	8	1.5	ns	-		
**13**	38	16.1	54	10.4	0.0152	0.2280	1.65	1.06-2.59	**51**	20	8.5	44	8.5	ns	-		
**14**	11	4.7	40	7.7	ns				**52**	6	2.5	11	2.1	ns	-		
**15**	24	10.2	44	8.8	ns	-											

n = number of times the allele occurs; F = allele frequency; *P*-value = calculated by Fisher’s exact test; Pc-value = value of *P* corrected by Bonferroni correction; OR = odds ratio; 95%CI = 95% confidence interval; ns= not significant (*p* > 0.05). This table is restricted to representative allele frequencies.

### Preliminary results from the other groups and subgroups versus the control group

Analysis of allele frequencies in subgroups 1B, 2A, 3A and group 3 versus the control group did not reveal any statistically significant differences in the HLA loci studied.

Comparison between group 2 and the control group showed that HLA-B*27 allele was more frequent in group 2 subjects (10.0% vs. 1.5% [*P* = 0.0180; *Pc* = 0.5580; OR = 7.1111; 95%CI = 1.7850 – 28.3286]) indicating a possible susceptibility to mucocutaneous ACL. Furthermore, the HLA-B*27 allele was more frequent in patients of subgroup 2B (25.0% vs. 1.5% [*P* = 0.0085; *Pc* = 0.2635; OR = 21.3333; IC 95% = 3.7218 - 122.2835]), suggesting susceptibility to mucocutaneous ACL.

HLA-A*01 was more frequent in patients from subgroup 3B (28.6% vs. 9% [*P* = 0.0360; *Pc* = 0.6480; OR = 4.0255; 95%CI = 1.2153 – 13.3341]), as was HLA-B*52 (21.4% vs. 2.1% [*P* = 0.0044; *Pc* = 0.1364; OR = 12.6198; 95%CI = 3.0827 – 51.6619]), suggesting possible susceptibility to relapse of cutaneous ACL at a location remote from the site of previous infection.

HLA-B*49 was more frequent in group 4 patients than in controls (9.1% vs. 1.5% [*P* = 0.0101; *Pc* = 0.3131; OR = 6.4000; 95%CI = 1.8472 – 22.1743]), indicating susceptibility to recurrent ACL.

After correction of *P*-values, we could not detect any association between susceptibility to or protection against ACL within the allele frequencies of MHC class I (HLA-A and HLA-B) and class II (HLA-DRB1) genetic markers among the several the patient and control groups and subgroups analyzed in this study. Table 5 shows all alleles with trends toward increased susceptibility and/or resistance to ACL in these groups and subgroups.

**Table 5:** Contribution of Class I and II HLA alleles to development of ACL in a Southern Brazilian population

**Allele**	**ACL**		**Controls (n = 260)**		***P*-value**	***P*c-value**	**OR**	**95%CI**
	**n**	***F *%**	**n**	***F %***				
**ACL overall (n = 169)**								
HLA-B*45	0	-	12	2.3	0.0046	0.1426	-	-
HLA-DRB1*13	51	15.1	54	10.4	0.0431	0.6465	1.53	1.01-2.31
**ACL (group 1; n = 136)**								
HLA-DRB1*13	44	16.2	54	10.4	0.0228	0.3420	1.66	1.08-2.55
HLA-B*35	53	19.5	68	13.1	0.0218	0.6758	1.67	1.08-2.29
HLA-B*44	38	14.0	46	8.9	0.0290	0.8990	1.67	1.05-2.64
HLA-B*45	0	-	12	2.3	0.0107	0.3317	-	-
**ACL (group 1A; n = 118)**								
HLA-B*44	35	14.8	46	8.9	0.0160	0.4969	1.79	1.12-2.87
HLA-B*45	0	-	12	2.3	0.0231	0.0225	-	-
HLA-DRB1*13	38	16.1	54	10.4	0.0152	0.2280	1.65	1.06-2.59
**ACL (group 2; n = 15)**								
HLA-B*27	3	10.0	8	1.5	0.0180	0.5580	7.11	1.78-28.33
**ACL (group 3B; n = 7)**								
HLA-B*52	3	21.4	11	2.1	0.0044	0.1360	12.61	3.08-51.66
**ACL (group 4; n = 22)**								
HLA-B*49	4	9.1	8	1.5	0.0101	0.3131	6.4	1.8-22.17

n = number of times the allele occurs; F = Allele frequency; *P*-value = calculated by Fisher’s exact test; *P*c-value = value of *P* corrected by Bonferroni correction; OR = odds ratio; 95%CI = 95% confidence interval.

## Discussion

One of the first studies assessing the potential association between HLA and cutaneous leishmaniasis involving serological methods was undertaken in France, with *L. guyanensis*. The study revealed a low HLA-Cw7 frequency was associated with pathogenesis of cutaneous leishmaniasis [16]. A Mexican study involving *L. mexicana* was undertaken by the molecular biology method, with 65 cases of cutaneous leishmaniasis and 100 controls. Whereas the frequencies of two alleles were associated with the protection against cutaneous leishmaniasis, namely HLA-DR2 (OR = 0.15; 95%CI = 0.05 – 0.38; *P* = 0.0000018) and HLA-DPB1*0401 (OR = 0.37; 95%CI = 0.21 – 0.67; *P* = 0.0004), two others were associated with increased susceptibility: HLA-DRB1*0407 (OR = 2.14; 95%CI = 1.34 – 3.40; *P* = 0.001) and HLA-DPA1*0401 (OR = 10.77; 95%CI = 1.25 – 80.73; *P* = 0.003) [20].

There have been few Brazilian studies on the association between HLA and susceptibility to/protection from cutaneous and mucocutaneous leishmaniasis [16-19], and there is no evidence of any studies in the literature addressing the association between HLA and ACL caused by *L. braziliensis* in the northwestern region of Paraná, southern Brazil. Studies of the association between HLA and visceral leishmaniasis are underway [20,21]; however, this is another form of the disease caused by another *Leishmania* species and presenting with different clinical manifestations. Thus, we chose not to compare the genetic markers found in these surveys with the results of the present study.

A serological study in Brazil involving *L. braziliensis* found that allele HLA-DQw3 was associated with risk of infection and HLA-DR2 was associated with protection (*Pc* = 0.004; RR = 0.007) against mucocutaneous leishmaniasis [18]. The present investigation found no associations with HLA-DR2 in any group.

Preliminary results of the allele frequency of HLA-B*27 (*P* = 0.0180, *Pc* = 0.5580) showed a trend toward susceptibility to ACL among patients with the mucocutaneous form. Although the patient samples were too small to draw any definitive conclusions, this finding corroborates that of Petzl-Erler et al. (1991), who analyzed 43 subjects with mucocutaneous leishmaniasis and compared them with 111 controls and initially found the HLA-B27 to be significantly associated (*P* = 0.029), but the significance did not hold after *P*-value correction.

Allele frequency of HLA-DRB1*13 (*P* = 0.0228, *Pc* = 0.3317) was conspicuous only in the non-recurrent cutaneous leishmaniasis groups, whereas HLA-B*49 was prominent in the recurrent disease group.

Studies have shown that CD4 + T lymphocytes of the T_h_1 type play a more important role in the immune response to mucosal disease, as there is a major predominance of CD8+ T lymphocytes in mucocutaneous lesions caused by *L. braziliensis*[22,23]. The present study has detected a trend toward association with an MHC class I allele (HLA-B*27) in patients with mucocutaneous leishmaniasis, which presents parasite antigens for CD8+ lymphocytes T. It may thus be surmised that a role of CD8+ T lymphocytes in mucocutaneous leishmaniasis cannot be ruled out.

An immunocytochemical study conducted in the Amazon region found that CD8+ T cells occurred at a higher level in all forms of the disease except mucocutaneous leishmaniasis, and included cases of cutaneous lesions caused by *L. braziliensis.* Its results seem to corroborate the role of CD8+ T cells in a balanced immune response to cutaneous lesions and, probably, in the healing process [23]. In the present study, HLA-B*45 was the only allele associated with a trend toward protection from cutaneous lesions. This corroborates the findings of Silveira *et al*., 2004, who endorse the role of CD8+ T lymphocytes in the healing of cutaneous lesions. Furthermore, HLA-B*45 involvement suggests that the common sequence for the antigen group may anchor parasite peptides and trigger a protecting response. It bears noting that HLA-B*45 is a rare allele in Brazilian populations, with frequencies ranging from 1.0 % to 1.6% in the state of Paraná [24]. However, its frequency in the control and patient groups was 2.3% and 0% respectively. Special attention should be given to this allele in ACL studies.

The frequency of the HLA-A*02-B*44-DRB1*07 and HLA-A*24-B*35-DRB1*01 haplotypes may be involved in susceptibility to cutaneous leishmaniasis. There is evidence in the literature of the involvement of these haplotypes with ACL. Studies have shown occurrence of the HLA-A*02-B*44-DRB1*07 haplotype in populations in the Brazilian states of Maranhão and even in the north of Paraná [25,26].

Research on the epidemiological profiles of ACL shows that infected patients are predominantly male [7,27]. Although the present study did find a male predominance in the infected group, no significant difference between the sexes was found in the control group. Even though males and females are exposed to the same environmental risk factors necessary for ACL acquisition, males are infected with the parasite much more often. The infrequency of ACL in females may be due to their use of repellents to protect themselves during the day and to their relatively infrequent exposure to risk factors, such as being near rivers and in the woods, as compared with men. Furthermore, the sample collection period for the control group may have influenced the number of women in the control group, as women were more likely than men to be home in the morning and afternoon.

In the present study, analysis of MHC class I (HLA-A and HLA-B) and class II (HLA-DRB1) genetic markers failed to show any association with susceptibility or protection to ACL after *P*-value correction. However, all results which initially had significant *P*-values should be carefully observed in further studies, and future studies be conducted with other markers. It bears stressing that, despite the very small sample size of some groups, our findings demonstrate that associations may, in fact, exist between HLA alleles and ACL, and that studies with larger sample sizes are warranted.

## Conclusions

Since the Brazilian population is highly miscegenated, results from studies undertaken in a single region should not be generalized to the whole country. Populations of several other regions should be researched and their allele diversity analyzed so that new associations can be identified or those already known corroborated. Such knowledge shall contribute towards future prophylactic and therapeutic interventions in Brazilian groups at higher risk of developing ACL.

## Abbreviations

HLA: Human Leukocyte Antigen; MHC: Major Histocompatibility Complex; PCR-SSO: Polymerase chain reaction with sequence-specific oligonucleotides; ACL: American cutaneous leishmaniasis; L. braziliensis: *Leishmania (Viannia) braziliensis*; F: Allele frequency; HW: Hardy –Weinberg equilibrium.

## Competing interest

The authors have no conflicts of interest to declare.

## Authors’ contributions

RSRC. sample collection; HLA typing; writing of the manuscript. RAD. sample collection; HLA typing. SMCG. - sample collection. JWV. statistical analyses. LMVC. laboratory tests for ACL; writing and revision of the manuscript. BSD. HLA typing; writing and revision of the manuscript STGV. laboratory tests for ACL; writing and revision of the manuscript. All authors have read and approved the submitted version of the manuscript.

## Pre-publication history

The pre-publication history for this paper can be accessed here:

http://www.biomedcentral.com/1471-2334/13/198/prepub
